# Factors Associated with Bothersome Lower Urinary Tract Symptoms in Middle-Aged Men Receiving Health Checkup

**DOI:** 10.1038/s41598-018-37605-4

**Published:** 2019-01-29

**Authors:** Teng-Kai Yang, Chi-Chih Chang, Hong-Chiang Chang, Hung-Ju Yang, Kuo-How Huang

**Affiliations:** 1 Department of Surgery, Yonghe Cardinal Tien Hospital, New Taipei City, Taiwan; 2 Department of Urology, National Taiwan University Hospital, Taipei, Taiwan; 3 The Interdisciplinary Nanoscience Centre, Aarhus University, Aarhus, Denmark

## Abstract

The prospective study is to investigate the associations between serum testosterone levels and LUTS among middle-aged men ≥40 years receiving health check-up. Lower urinary tract symptoms were evaluated by the self-administered International Prostate Symptom Score questionnaire. Serum prostate specific antigen and total testosterone level were checked in all subjects. A total of 1752 men were enrolled into the study. The mean age was 55.6 ± 9.7 years. All study subjects were stratified into low, medium and high testosterone levels by two cut-off, 3.0 and 4.11 ng/mL. We found that testosterone levels were significantly associated with metabolic syndrome and body fat components. Compared to those with low testosterone levels, subjects with high and medium testosterone had a significantly higher IPSS (5.84 ± 5.55 vs 6.71 ± 5.68 and 6.34 ± 5.66, p = 0.032) and storage score (2.76 ± 2.29 vs 3.20 ± 2.49 and 2.90 ± 2.49; *p* = 0.009), and a more moderate/severe LUTS (IPSS ≧ 8) (26.5% vs 35.7% and 29.9%; p = 0.002). Multivariate analyses showed that high vs low testosterone levels (OR, 1.76; 95% CI, 1.26–2.45) and prostate volume ≧25 vs <25 mL (OR, 1.38; 95% CI, 1.04–1.82) significantly associated with the presence of moderate/severe LUTS. Pearson correlation analyses showed significantly positive correlations between testosterone level and IPSS in whole study sample (Pearson correlation coefficient, 0.066; p < 0.01) and in the subgroup of moderate/severe LUTS (Pearson correlation coefficient, 0.038; p < 0.05). In conclusion, high testosterone and prostate volume adversely impacted LUTS in our target population.

## Introduction

Lower urinary tract symptoms (LUTS) are increasingly prevalent in middle-aged and old men. LUTS of the majorities of middle-aged men are usually attributed to benign prostatic enlargement (BPE) and may be associated with metabolic syndrome (MS)^1^. However, BPE and MS are also prevalent diseases in the middle-aged men, so the association with LUTS is questionable: is it a real association or simply an epiphenomenon. In addition, several putative hormonal pathways (such as testosterones) are associated with BPE and MS^2^.

To date, the correlation between LUTS and testosterone in middle-aged men remains controversial. Emerging evidence indicate a positive correlation between testosterone and LUTS in men^3^. Serum testosterone affects metabolic factors, where LUTS occurs through autonomic nerve dysfunction and inflammation^4^. Crawford *et al*. indicated that worsening of LUTS following testosterone replacement therapy in hypogonadal men may be due to stimulation of prostatic cells previously deprived of testosterone^5^. It is widely accepted that severe LUTS is one of relative contraindications for testosterone replacement therapy for fear of symptom exacerbation^6,7^. Recently, one systemic review reported that testosterone is not independently associated with either the pathogenesis or severity of LUTS^8^. Another study demonstrated that the impact of testosterone replacement therapy on LUTS were similar to the placebo effect^9^. On the contrary, previous study reported that bioactive testosterone benefit LUTS^10^. Testosterone replacement may reduce LUTS for selected groups with late-onset hypogonadism^11,12^.

Since there existed discrepancies about the impact of testosterone level on LUTS, we sought to clarify the associations between serum testosterone level and LTUS in in middle-aged men.

## Materials and Methods

### Study population

In this prospective study, we enrolled consecutive subjects aged 40 years or more (range, 40 to 65) who voluntarily underwent self-paid medical check-up at the Health Management Center of National Taiwan University Hospital between January 2010 and December 2014. Prior to the examination, a self-administered questionnaire was used to collect information on the demographic characteristics, social habits, health and medical histories, clinical symptoms and LUTS of the participants. LUTS were evaluated using a validated Chinese version of the international prostatic symptoms score (IPSS) questionnaire. Subjects were excluded from the study if any one of the following applied to them: (1) used hormone replacement therapy or 5-alpha-reductase inhibitors within the last 6 months, (2) had a history of prostate cancer or underwent prostate surgery, and (3) is currentlyon medications known to interfere with voiding symptoms such as anticholinergics, sympathomimetics, and sympatholytics. This study was approved by the institutional review board at National Taiwan University Hospital (No. 201407108RINC). All methods were performed in accordance with the relevant guidelines and regulations of the institution. The written informed consent forms from all subjects who met the inclusion criteria were obtained. Basic clinical and laboratory data such as age, height, weight, body mass index, blood pressure, waist circumference, serum biochemistry profiles, prostate specific antigen (PSA) and total testosterone levels were collected. Additionally, all participants underwent uroflowmetry testing and specially trained nurses recorded the results. Transrectal ultrasound and digital rectal examination were performed by experienced urologists to evaluate prostatic volume (PV), shape, symmetry, firmness and nodularity.

### Definition of MS

According to the National Cholesterol Education Program – Adult Treatment Panel III^13^. MS in men is defined by the presence of three or more of the following risk factors: (1) waist circumference > 90 cm (for Asian population), (2) fasting blood sugar > 100 mg/dl or previously diagnosed type 2 diabetes, (3) serum triglyceride level > 150 mg/dl, 4) systolic blood pressure > 130 mmHg or diastolic blood pressure > 85 mm Hg, and 5) HDL cholesterol < 40 mg/dl.

### Statistics

For descriptive findings, the continuous variables were presented as mean and standard deviations (SD), and categorical variables as percentages. The total testosterone level below 3 ng/mL has been used in clinical practice as a cut-off for the diagnosis of low testosterone and testosterone deficiency. All study subjects were stratified into three subgroups based on serum total testosterone as low, medium, and high testosterone levels by using 3.0 and 4.11 ng/mL as cut-off points. In our study, the range of testosterone level in the low testosterone subgroup was 0.2–3.0 ng/mL. For subjects with serum testosterone level >3 ng/mL, we divided them into medium (range 3.1–4.1 ng/mL) and high (>4.1 ng/mL) testosterone subgroups for further analyses. Differences in baseline characteristics among the three groups were analyzed using one-way ANOVA and chi-squared tests. A *p*-value less than 0.05 was considered statistically significant. Multivariate analyses to evaluate the impact of the respective factors on LUTS were performed by logistic regression method. All potential risk factors for LUTS such as age, prostate volume, serum PSA level and the presence of metabolic syndrome were included in the analyses. To examine the correlations between testosterone level as continuous parameter and other parameters, we calculated Pearson correlation coefficients to measure the linear associations of testosterone level with IPSS, age, serum PSA and prostate volume. In addition, we divided the cohort into two groups, mild (IPSS < 8) and moderate/severe LUTS (IPSS ≥ 8). We analyzed the relationships of testosterone level with IPSS, age, serum PSA and prostate volume in subjects with moderate/severe LUTS. All analyses were performed with SPSS statistical software for Windows (SPSS, version 13.0; SPSS Inc., Chicago, IL).

## Results

Of the 2083 men who volunteered to undergo medical check-up during the study period, we excluded 331 subjects. Patients were excluded if any one of the exclusion criteria applied: the use of hormone replacement therapy (n = 13) or 5-alpha reductase inhibitors (n = 56) within the last 6 months, having a history of prostate cancer (n = 13) or prostate surgery (n = 36), or current use of anticholinergics (n = 97) or sympatholytics (n = 135). The remaining 1752 men were included in the study. Table 1 lists the baseline characteristics of all study subjects. The mean testosterone level was 3.76 ng/mL (SD 1.38). Overall, 138 (7.8%) of the study subjects had hypogonadism (testosterone level below 2.30 ng/mL). All study subjects were stratified into low, medium and high testosterone subgroup by cut-off of 3.0 and 4.11 ng/mL.

**Table 1:** Baseline characteristics, metabolic syndrome parameters and body mass indices in subjects stratified by low, medium and high serum testosterone level.

	Low (n = 591)	Medium (n = 578)	High (n = 583)	*p* value
Testosterone (ng/ml)	2.43 ± 0.52	3.58 ± 0.28	5.27 ± 1.14	<0.001
Age (year)	53.3 ± 6.63	53.1 ± 6.18	53.5 ± 6.42	0.60
Height (cm^2^)	170 ± 9.50	170 ± 6.00	170 ± 6.06	0.49
Weight (kg)	76.0 ± 12.3	72.2 ± 9.47	70.2 ± 9.01	<0.001
BMI (kg/m^2^)	26.3 ± 3.60	25.0 ± 2.85	24.2 ± 2.77	<0.001
PV (ml)	27.0 ± 10.1	27.0 ± 10.1	26.5 ± 10.7	0.70
Serum PSA	1.22 ± 1.52	1.23 ± 1.51	1.30 ± 1.38	0.60
Serum Albumin	4.73 ± 0.25	4.68 ± 0.26	4.63 ± 0.24	<0.001
Serum HbA1c	5.92 ± 0.83	5.75 ± 0.66	5.70 ± 0.69	<0.001
Serum Cholesterol	199 ± 43.0	198 ± 33.7	197 ± 33.5	0.66
Serum Uric Acid	6.70 ± 1.40	6.49 ± 1.18	6.27 ± 1.23	<0.001
Metabolic syndrome	308 (52.1)	216 (37.4)	135 (23.2)	<0.001
Raised WC	353 (59.7)	270 (46.8)	220 (37.7)	<0.001
High blood pressure	351 (59.4)	322 (55.8)	282 (48.4)	0.001
High fasting sugar	231 (39.1)	169 (29.2)	131 (22.5)	<0.001
High triglyceride	276 (46.7)	180 (31.1)	122 (20.9)	<0.001
Low HDL	294 (49.7)	229 (39.6)	170 (29.2)	<0.001
Body mass indices				
Body fat percent (%)	27.2 ± 4.49	25.2 ± 4.45	24.1 ± 4.44	<0.001
Fatness (kg)	18.6 ± 15.8	12.4 ± 12.9	8.78 ± 12.0	<0.001
Body fat mass (kg)	21.1 ± 6.30	18.5 ± 5.17	17.1 ± 4.89	<0.001
Lean body mass (kg)	54.9 ± 6.80	53.7 ± 5.43	52.9 ± 5.70	<0.001
Total body water (kg)	39.5 ± 4.89	38.7 ± 3.90	38.1 ± 4.10	<0.001
Basal metabolic rate	1599 ± 153	1566 ± 116	1544 ± 132	<0.001
Intracellular fluid (kg)	26.4 ± 3.26	25.8 ± 2.61	25.4 ± 2.74	<0.001
Extracellular fluid (kg)	13.2 ± 1.63	12.9 ± 1.30	12.7 ± 1.37	<0.001
Protein (kg)	11.0 ± 1.56	10.7 ± 1.65	10.7 ± 1.56	0.007
Muscle (kg)	50.2 ± 7.43	48.8 ± 7.58	48.4 ± 7.17	<0.001
Mineral (kg)	4.32 ± 0.79	4.08 ± 0.71	3.99 ± 0.66	<0.001

PV: prostate volume; TG = serum triglyceride; BP = blood pressure; WC = waist circumference; FBG: fasting blood sugar.

The mean age was 55.6 years (SD 9.7). Age, height, prostate volume and serum PSA did not differ significantly among the three groups. Contrastingly, weight (76.0 ± 12.3 vs. 72.2 ± 9.47 and70.2 ± 9.01 kg) and body mass index, (26.3 ± 3.60 vs. 25.0 ± 2.85and 24.2 ± 2.77 kg/m^2^) of subjects in the low testosterone level group were significantly higher compared to those in the medium and high testosterone groups (*p* < 0.001 for both). Several metabolism-related blood chemistry profiles were significantly higher in the low testosterone group compared to the medium and high testosterone groups, including serum albumin (4.73 ± 0.25 vs. 4.68 ± 0.26 and 4.63 ± 0.24), glycated hemoglobin (5.92 ± 0.83 vs. 5.75 ± 0.66 and 5.70 ± 0.69) and uric acid (6.70 ± 1.40 vs. 6.49 ± 1.18 and 6.27 ± 1.23). Similarly, five MS associated medical conditions were more prevalent in the low testosterone group compared to the medium and high testosterone groups (all *p* < 0.001); these include an increase in waist circumference (59.7% vs. 46.8% and 37.7%), higher blood pressure (59.4% vs. 55.8% and 48.4%), higher fasting blood sugar (39.1% vs. 29.2% and 22.5%), higher triglyceride levels (46.7% vs. 31.1% and 20.9%) and higher high-density lipoprotein (HDL) levels (49.7% vs 39.6% and 29.2%). Additionally, 52.1%of subjects in the low testosterone group fit the diagnosis of MS compared to 37.4% and 23.2% in the medium and high testosterone groups, respectively (*p* < 0.001). Body mass indices such as body fat (%), fatness (kg), body fat mass (kg), lean body mass (kg), total body water (kg), basal metabolic rate, intracellular fluid (kg), extracellular fluid (kg), protein (kg), muscle (kg) and mineral (kg) were significantly different in the low testosterone group compared to the medium and high testosterone groups (all p < 0.05). Subjects with low testosterone levels had significantly higher body fat percentage (27.2 ± 4.49 vs. 25.2 ± 4.45 and 24.1 ± 4.44%), fatness (18.6 ± 15.8 vs.12.4 ± 12.9 and 8.78 ± 12.0 kg) and body fat mass (21.1 ± 6.30 vs. 18.5 ± 5.17 and 17.1 ± 4.89 kg) and basal metabolic rate (1599 ± 153 vs 1566 ± 116 and 1544 ± 132) compared to those with medium and high testosterone levels (all *p* < 0.001).

In Table 2, we compared LUTS across the three groups. Subjects with low testosterone levels had lower IPSS scores than subjects with medium and high testosterone levels (5.84 ± 5.55 vs 6.34 ± 5.66 and 6.71 ± 5.68; *p* = 0.032). Moreover, subjects with low testosterone levels had significantly lower storage scores than subjects with medium and high testosterone levels (2.76 ± 2.29 vs 2.90 ± 2.49 and 3.20 ± 2.49; *p* = 0.009). Subjects with high testosterone levels had more severe symptoms of frequency and intermittency (*p* < 0.05). Overall, 35.7% of subjects in the high testosterone group had moderate/severe LUTS (IPSS ≧ 8), compared to 29.9% in the medium testosterone group and 26.5% in the low testosterone group.

**Table 2:** LUTS of study subjects stratified by testosterone level.

	Low (n = 571)	Medium (n = 562)	High (n = 558)	*p* value
Total IPSS	5.84 ± 5.55	6.34 ± 5.66	6.71 ± 5.68*	0.032
LUTS severity n (%)				0.002
Mild (IPSS 0–7)	420 (73.6)	394 (70.1)	359 (64.3)	
Moderate (IPSS 8–19)	130 (22.8)	145 (25.8)	184 (33.0)	
Severe (IPSS ≧ 20)	21 (3.7)	23 (4.1)	15 (2.7)	
Storage score	2.76 ± 2.29	2.90 ± 2.49	3.20 ± 2.49*	0.009
Frequency	1.30 ± 1.27	1.39 ± 1.33*	1.60 ± 1.44*	0.001
Urgency	0.56 ± 0.91	0.62 ± 0.96	0.65 ± 0.97	0.26
Nocturia	0.90 ± 0.81	0.90 ± 0.86	0.95 ± 0.84	0.53
Voiding score	3.08 ± 3.88	3.43 ± 3.83	3.52 ± 3.90	0.13
Incomplete emptying	1.05 ± 1.29	1.19 ± 1.39	1.16 ± 1.27	0.15
Intermittency	0.75 ± 1.20	0.92 ± 1.30*	0.92 ± 1.29*	0.031
Weak stream	0.81 ± 1.33	0.81 ± 1.25	0.93 ± 1.38	0.22
Straining	0.47 ± 0.94	0.51 ± 0.93	0.51 ± 0.97	0.78
Qmax < 15 ml/sec, n (%)	149(26.1)	168(29.9)	137(24.6)	0.12

We further subdivided subjects into three groups within age categories of 40–49, 50–59, and 60–69 years. We then compared the MS and LUTS among three groups. As shown in Table 3, subjects in older age categories had higher IPSS scores compared to those in younger group. There was no significantly difference for MS diagnosis among three groups; nevertheless, several MS associated medical conditions such as blood pressure, fasting blood sugar and serum triglyceride were significantly different among three groups (all p < 0.001). Several body mass indices such as lean body mass (kg), total body water (kg), basal metabolic rate, intracellular fluid (kg), extracellular fluid (kg), protein (kg), muscle (kg) and mineral (kg) were significantly different among three groups (all p < 0.001).

**Table 3:** IPSS, metabolic syndrome parameters and body mass indices in study subjects stratified by age.

Age	40–49 (n = 520)	50–59 (n = 889)	60–65 (n = 343)	*p valule*
Testosterone (ng/ml)	3.76 ± 1.35	3.77 ± 1.36	3.73 ± 1.49	0.93
Total IPSS	5.13 ± 4.82	6.43 ± 5.77	7.71 ± 6.08	<0.001
Metabolic syndrome	183 (35.3)	331 (37.2)	145 (42.3)	0.11
Raised WC	244 (46.9)	431 (48.5)	168 (49.0)	0.84
High blood pressure	247 (47.6)	498 (56.0)	210 (61.2)	<0.001
High fasting sugar	87 (16.7)	295 (33.2)	149 (43.4)	<0.001
High triglyceride	208 (40.0)	287 (32.3)	83 (24.2)	<0.001
Low HDL	219 (42.1)	339 (38.1)	135 (39.4)	0.34
Body mass indices				
Body fat percent (%)	25.5 ± 4.80	25.5 ± 4.41	25.8 ± 4.59	0.40
Fatness (kg)	13.5 ± 15.2	12.9 ± 13.9	14.3 ± 13.6	0.30
Body fat mass (kg)	19.4 ± 6.15	18.8 ± 5.47	18.7 ± 5.57	0.15
Lean body mass (kg)	55.1 ± 6.07	53.9 ± 5.64	52.2 ± 5.27	<0.001
Total body water (kg)	39.7 ± 4.37	38.8 ± 4.06	37.6 ± 3.80	<0.001
Basal metabolic rate	1595 ± 130	1570 ± 122	1539 ± 122	<0.001
Intracellular fluid (kg)	26.4 ± 2.91	25.9 ± 2.71	25.1 ± 2.53	<0.001
Extracellular fluid (kg)	13.2 ± 1.46	12.9 ± 1.35	12.5 ± 1.27	<0.001
Protein (kg)	11.1 ± 1.50	10.8 ± 1.54	10.4 ± 1.60	<0.001
Muscle (kg)	50.4 ± 7.05	49.2 ± 7.14	47.4 ± 7.46	<0.001
Mineral (kg)	4.24 ± 0.72	4.13 ± 0.71	4.01 ± 0.73	<0.001

PV: prostate volume; TG = serum triglyceride; BP = blood pressure; WC = waist circumference; FBG: fasting blood sugar.

Table 4 show the potential predictors of moderate/severe LUTS (IPSS ≧ 8) in all study subjects. In univariate models, the testosterone levels (OR, 1.54; 95% CI, 1.20–1.99), age ≧ 53 vs. <53 years (OR, 1.49; 95% CI, 1.21–1.83) and prostate volume ≧ 25 mL vs. <25 mL (OR, 1.43; 95% CI, 1.10–1.86) were significantly associated with the risk of moderate/severe LUTS. In line with this, multivariate analysis showed that the testosterone levels (OR, 1.76; 95% CI, 1.26–2.45), and prostate volume ≧ 25 mL vs <25 mL (OR, 1.38; 95% CI, 1.04–1.82) were independent predictors of bothersome LUTS.

**Table 4:** Factors associated with moderate/severe LUTS (IPSS ≧ 8) in all subjects after univariate and multivariate analyses.

		Univariate		Multivariate	
		OR	95% CI	OR	95% CI
Testosterone level	Low	1		1	
	Medium	1.19	(0.92–1.54)	1.36	(0.98–1.90)
	High	1.54*	(1.20–1.99)	1.76*	(1.26–2.45)
Age	<53	1		1	
	≧53	1.49*	(1.21–1.83)	1.20	(0.92–1.57)
Prostate volume	<25 mL	1		1	
	≧25 mL	1.43*	(1.10–1.86)	1.38*	(1.04–1.82)
PSA	<1.0 ng/dl	1			
	≧1.0 ng/dl	1.19	(0.97–1.47)	1.08	(0.82–1.43)

Table 5 shows the correlations between testosterone level as a continuous parameter and other parameters such as age, prostate volume, PSA and IPSS in all study subjects. The results showed there was significantly positive correlation between testosterone level and IPSS (Pearson correlation coefficient, 0.066; p < 0.01). We then divided the cohort into two groups, mild (IPSS < 8) and moderate/severe LUTS (IPSS ≥ 8). We analyzed the relationships of testosterone level with IPSS, age, serum PSA and prostate volume in subjects with moderate/severe LUTS. As is shown in Table 6, testosterone level was positively correlated with LUTS (Pearson correlation coefficient, 0.038; p < 0.05) in subjects with moderate/severe LUTS.

**Table 5:** Pearson correlation coefficients among testosterone level, age, prostate volume, PSA and IPSS.

	Testosterone level	Age	Prostate volume	PSA	IPSS
Testosterone level	1	−0.08	−0.013	0.014	0.066**
Age	−0.08	1	0.255**	0.156**	0.180**
Prostate volume	−0.013	0.255**	1	0.382**	0.198**
PSA	0.114	0.156**	0.382**	1	0.12**
IPSS	0.066**	0.180**	0.198**	0.12**	1

**p < 0.01.

**Table 6:** Pearson correlation coefficients among testosterone level, age, prostate volume, PSA and IPSS in subjects with moderate/severe LUTS.

	Testosterone level	Age	Prostate volume	PSA	Bothersome LUTS
Testosterone level	1	−0.021	−0.041	−0.014	0.038*
Age	−0.021	1	0.235**	0.209**	0.147**
Prostate volume	−0.041	0.235**	1	0.394**	0.198**
PSA	−0.014	0.209**	0.394**	1	0.100**
Bothersome LUTS	0.038*	0.147**	0.198**	0.100**	1

*p < 0.05, **p < 0.01.

## Discussion

The significant association between MS and low testosterone levels has been well studied. Testosterone levels have been reported to be a valuable indicator of MS in middle-aged men^14^. Additionally, there is an inverse relationship between testosterone and body fat in men^15^. Our results confirmed the significant associations between testosterone levels and MS as well as body fat components in the present study.

LUTS are mostly attributed to BPE in middle-aged men and declining testosterone levels and the presence of MS are prevalent in middle-aged men^16,17^. These two important factors may also play a role in LUTS of middle-aged men. MS has been thought to be associated with prostate enlargement^18,19^. The correlations between severity of male LUTS and MS-related medical conditions have been reported^20–23^. Nevertheless, the exact relationship between MS and LTUS remains controversial. Some studies indicated a positive correlation between MS and LUTS^24,25^. Several studies from Asia papulation suggested no significant associations between MS and male LUTS^25–27^. In contrast, some studies indicated that men with MS had lower risk of developing moderate to severe LUTS compared to those without MS^28^. Eom *et al*. reported that MS and accompanying hyperinsulinemia may have a favorable impact on IPSS and voiding score^29^.

The relationship among testosterone, LUTS and MS is ambiguous. Cohan *et al*. introduced a hypogonadism-obesity-BPE-LUTS model and found that abdominal obesity and intra-abdominal pressure caused by the net positive caloric balance leads to reduced testosterone and elevated the estrogen levels. The continuous hypogonadism will enlarge the prostate and worsen LUTS^30^. An animal model showed that hypercholesterolemia and the associated pelvic ischemia can cause bladder fibrosis, smooth muscle atrophy, and decrease bladder compliance^31^. Long-term testosterone replacement in men with hypogonadism and erectile dysfunction can restore functions, reduce obesity parameters and improve metabolic syndrome and health-related quality of life^32^. Our results show that larger prostate volume and high testosterone levels were significantly associated with moderate/severe LUTS (IPSS ≧ 8) in our target male population. In contrast, age, metabolic syndrome and metabolic syndrome-associated medical conditions were not independent predictors of moderate/severe LUTS in our study sample.

Several studies also showed a negative correlation between testosterone levels and LUTS^33,34^. However, most of these study cohorts were composed of men with LUTS or erectile dysfunction seeking for help. Antunes *et al*. reported that higher testosterone levels are associated with a severe AUA score symptom index in obese patients^35^. The recommendations for testosterone replacement therapy made by the Endocrine Society were critically re-examined recently^36^; it was suggested that men with large prostates should be treated with caution. Based on the results of our study, testosterone replacement therapy should be approached cautiously in middle-aged men with severe LUTS. Recent studies reported that testosterone replacement therapy did not induce aggravation of LUTS^8,9,37^. Kohn *et al*. reviewed a series of randomized control trials and concluded the impacts of testosterone replacement therapy on LUTS were similar to placebo effect^9^. The results of this study was limited by the characteristics of study participants. The study did not include men with severe LUTS, who were the main concern in prescribing testosterone replacement^38^. Since the problem remains unsolved, patients with LUTS should be treated carefully regarding testosterone replacement therapy.

Our study has several strengths. Firstly, all study subjects excluded those treated with BPE-related medications and diagnosed with prostate disease. Moreover, all study subjects were enrolled in a screening setting. The bias from BPE or prostate disease was minimized. Secondly, we collected comprehensive anthropometric measurements and evaluations on metabolic syndrome, LUTS, uroflowmetry, transrectal ultrasound and digital rectal examination, which allowed us to elucidate the said mutual relationships. This work is more difficult to achieve in a large-scale population-based study. This study provides a valuable link between metabolic disorders, testosterone levels and LUTS. The results provided important information on the pathogenesis of LUTS and therapeutic guidance of testosterone replacement therapy in such patients.

The present study has several limitations that must be considered when interpreting the results. Firstly, the present study is cross-sectional in nature and the effect of time was not considered. Several risk factors, such as the subjects’ life styles and socioeconomic statuses, were not collected and analyzed^20,26,39,40^. Secondly, only ethnic Taiwanese individuals who attended the voluntary annual medical health check-up were enrolled into the study. Nevertheless, the prevalence was 29.6% (518/1752) for moderate/severe LUTS and 37.6% (659/1752) for metabolic syndrome. These results were comparable to those of the population-based study in Taiwan^41,42^. We should beware of the limitations when extrapolating the results to other populations. Third, the data of dihydrotestosterone levels were lacking in this study. The measurement of dihydrotestosterone may provide more information in this study, especially considering the effect of dihydrotestosterone on prostate enlargement. More evidence with larger samples and a long-term follow up is necessary to clarify the associations between LUTS with testosterone and metabolic syndrome in different male populations at various ages.

## Conclusion

In the present study, we elucidated a significant impact of total serum testosterone levels and prostate volume on moderate/severe LUTS among middle-aged men receiving health checkup. Testosterone supplement therapy should be used cautiously in patients with moderate/severe LUTS and large prostates.
